# Artificial intelligence insights into hippocampal processing

**DOI:** 10.3389/fncom.2022.1044659

**Published:** 2022-11-07

**Authors:** Hannah S. Wirtshafter, Matthew A. Wilson

**Affiliations:** ^1^Department of Biology, Massachusetts Institute of Technology, Cambridge, MA, United States; ^2^Picower Institute for Learning and Memory, Cambridge, MA, United States; ^3^Massachusetts Institute of Technology, Cambridge, MA, United States; ^4^Department of Brain and Cognitive Sciences, Massachusetts Institute of Technology, Cambridge, MA, United States

**Keywords:** hippocampus, replay, navigation, learning, context, DeepMind, muZero, model-based learning

## Abstract

Advances in artificial intelligence, machine learning, and deep neural networks have led to new discoveries in human and animal learning and intelligence. A recent artificial intelligence agent in the DeepMind family, muZero, can complete a variety of tasks with limited information about the world in which it is operating and with high uncertainty about features of current and future space. To perform, muZero uses only three functions that are general yet specific enough to allow learning across a variety of tasks without overgeneralization across different contexts. Similarly, humans and animals are able to learn and improve in complex environments while transferring learning from other contexts and without overgeneralizing. In particular, the mammalian extrahippocampal system (eHPCS) can guide spatial decision making while simultaneously encoding and processing spatial and contextual information. Like muZero, the eHPCS is also able to adjust contextual representations depending on the degree and significance of environmental changes and environmental cues. In this opinion, we will argue that the muZero functions parallel those of the hippocampal system. We will show that the different components of the muZero model provide a framework for thinking about generalizable learning in the eHPCS, and that the evaluation of how transitions in cell representations occur between similar and distinct contexts can be informed by advances in artificial intelligence agents such as muZero. We additionally explain how advances in AI agents will provide frameworks and predictions by which to investigate the expected link between state changes and neuronal firing. Specifically, we will discuss testable predictions about the eHPCS, including the functions of replay and remapping, informed by the mechanisms behind muZero learning. We conclude with additional ways in which agents such as muZero can aid in illuminating prospective questions about neural functioning, as well as how these agents may shed light on potential expected answers.

## Introduction

A goal of artificial intelligence research is to design programs that can solve problems as well as, or better than humans. Algorithms such as AlphaGo and MuZero can compete with and beat the best trained humans at tasks such as Chess, Go, and Atari (Silver et al., 2016; Schrittwieser et al., 2020). Other artificial intelligence programs can do image classification at accuracy on par with and speeds surpassing humans (Krizhevsky et al., 2012). Ideally, advances in artificial intelligence and machine learning would transfer to understanding human intelligence. However, this often fails to be the case as machines and humans use different mechanisms to learn. For instance, while a supervised image classifier can only distinguish between an elephant and a dog after being presented with 1,000s of images, a child can distinguish between these animals after only a few stimulus exposures (Krizhevsky et al., 2012; Zador, 2019).

However, advances in artificial intelligence, machine learning, and deep neural networks have also led to new discoveries in human and animal learning and intelligence (Richards et al., 2019). Notably, artificial intelligence research has proposed cellular and systems level mechanisms of reinforcement learning (Wang et al., 2018; Dabney et al., 2020), auditory processing (Kell et al., 2018; Jackson et al., 2021), object recognition (Cichy et al., 2016), and spatial navigation (Banino et al., 2018; Bermudez-Contreras et al., 2020; Lian and Burkitt, 2021). In several of these studies, artificial intelligence showed that specific inputs or parameters, such as grid cell coding, were sufficient to create a representation of the environment in which performance on a task closely resembles or surpasses human ability (Banino et al., 2018; Kell et al., 2018).

A recent artificial intelligence agent in the DeepMind family, muZero, can complete a variety of tasks with limited information about the world in which it is operating and with high uncertainty about features of current and future space (Schrittwieser et al., 2020). For instance, unlike in chess, where each move results in a predictable board layout, Atari games (as well as real-world scenarios) involve complex environments with unknown action consequences. MuZero can perform impressively in these environments using only three functions. These functions first encode the environment, then compute potential actions and rewards, and then finally predict an estimation of the future environment. These three functions are both general and specific enough to allow learning across a variety of tasks without overgeneralization across different contexts.

Like muZero, humans and animals can learn context dependent and independent task schema. Decisions by humans and animals are also made in complex spaces and result in unknown consequences, and, like muZero, humans and animals are still able to learn and improve in these environments. In particular, the mammalian extrahippocampal system (eHPCS) can guide spatial decision making while simultaneously encoding and processing spatial and contextual information (O’Keefe, 1976; Penick and Solomon, 1991; Hafting et al., 2005; Moser et al., 2008; Wirtshafter and Wilson, 2021). The eHPCS is also able to adjust contextual representations depending on the degree and significance of environmental changes and the significance of environmental cues (Hollup et al., 2001; Leutgeb et al., 2005; Lee et al., 2006; Fyhn et al., 2007; Wikenheiser and Redish, 2015b; Boccara et al., 2019; Wirtshafter and Wilson, 2020). In this opinion, we will argue that the muZero functions parallel those of the hippocampal system. We will show that the different components of the muZero model provide a framework for thinking about generalizable learning in the eHPCs.

## Parallels in muZero with hippocampal function can help explain mechanisms of context independent learning

The ability to generalize learning to unknown contexts is a key component of human and animal intelligence. For instance, a driver is able to generalize that a red light means stop, regardless of the location of the intersection, the car being driven, or the time of day. While the neural mechanisms behind generalization remain largely unknown, most artificial intelligence models generalize using either model-free or model-based learning. Model-free systems, which learn through experience, are inflexible, fast to run but slow to adapt to change, and estimate the best action based on cached values. Conversely, model-based systems, which aim to learn a model of the environment, are flexible but inefficient, highly sensitive to details of task representations, and easily adaptive to environmental changes (Stachenfeld et al., 2017; Gershman, 2018; Vértes and Sahani, 2019). Although model-based, muZero improves on many shortcomings of model-based and model-free systems by modeling only the elements of the environment important for decision making: the value (the strength of the correct position), the policy (the best action to take), and the reward (the benefit of the previous action). It models these three components using a representation function, a dynamics function, and a predictions function (Schrittwieser et al., 2020). Below, we argue these functions parallel functions found in the hippocampal system, and can be used to provide further insight into and build on current theories of hippocampal function during learning generalization.

## The representation function

The first step of muZero processing is to encode the actual environment to a hidden state using a representation function (Schrittwieser et al., 2020). This hidden state is a rendition of the environment that contains crucial information for performing the task. Similarly, several structures in the eHPCS flexibly represent environmental cues, with some regions placing emphasis on cues and locations of significance. Specifically, the entorhinal cortex (EC), CA regions of the hippocampus (HPC), and the lateral septum (LS) all contain cells whose firing rate is modulated by the location of the animal (termed grid cells, place cells, and “place-like” cells, respectively) (O’Keefe, 1976; Hafting et al., 2005; Moser et al., 2008; Wirtshafter and Wilson, 2019). Spatial representations in all three regions can be restructured by the presence of reward or cues/landmarks of interest, in which cells show a bias for coding for these significant locations (Hollup et al., 2001; Lee et al., 2006; Boccara et al., 2019; Wirtshafter and Wilson, 2020). When there are large changes to the environment, HPC and EC cell populations change representations and are said to “remap” (Fyhn et al., 2007; Colgin et al., 2008). It is likely that this phenomenon also occurs in the LS but it has not been studied.

**FIGURE 1:**
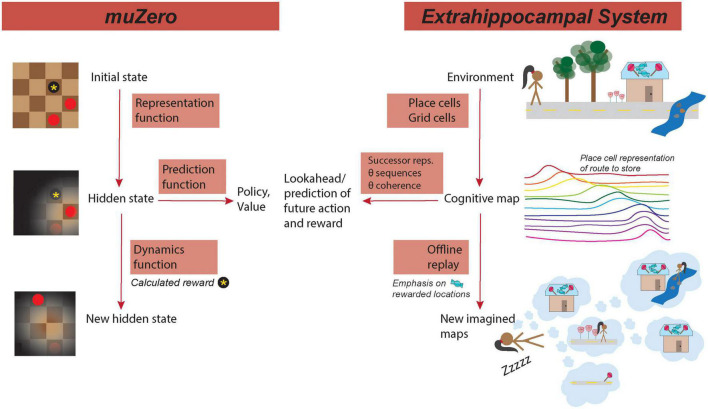
MuZero mirrors hippocampus processing. **(Left)** Functions of muZero. **(Right)** Analogous/similar hippocampal functions. **(Left top)** In muZero, the representation functions maps the initial state, such as the position on a checker board, to an internal hidden state that emphasizes important features of the environment. **(Right top)** A similar processing in the extrahippocampal system (EHPCs) represents the environment in a cognitive map as a serious of cell positions encoded by grid and place cells. Place cells tend to have fields clustered around the location of a reward, such as the destination of a candy shop. **(Left center)** From the hidden state, a prediction function computes potential actions (policies) and rewards (values) of the location in the current state. **(Right center)** In the EHPCs, place cells encode features of the environment in relation to their predictive relationship with other features, such as the potential presence of reward. Additionally, state prediction in HPC (*via* theta sequences) can be coupled through theta coordination coherence to other areas of the brain that process action and reward. **(Bottom left)** In muZero, the dynamics function calculates reward (such as a captured checkers piece) and computes a new hidden state. **(Bottom right)** In the EHPCs, offline replay, which occurs during periods of sleep, emphasizes locations of previous reward. During replay, events can be selectively sampled from visited event space, with priority given to representations or locations that correspond with reward. Reward-predictive representations can then be compressed into a lower dimensional space. Replay can be comprised of novel state configurations that have not been experienced, such as a novel route to the reward location.

Selectively coding and over-representing causal features of the environment is important for generalization of learning across contexts (Collins and Frank, 2016; Lehnert et al., 2020). The refinement of this coding allows the inference of critical salient environmental features that may allow or preclude generalization to other contexts—if the salient features are shared among two contexts, learning may be generalizable between the contexts (Collins and Frank, 2016; Abel et al., 2018; Lehnert et al., 2020). This muZero processing parallels the theory that the hippocampus can generalize across different contexts during learning by treating states that have equivalent actions or rewards as equivalent states. By compressing environmental representations into lower dimensionality abstractions, learning can be sped up and more easily transferred between environments with shared features (Lehnert et al., 2020). As such, the representation function provides a means by which learning can be generalized through contextual learning (Figure 1).

## The dynamics and prediction functions and the Monte Carlo tree search algorithm

In muZero, a simulation always begins with the representation of the current state (environment), as created using the representation function. From this initial state, a number of hypothetical new states and their corresponding potential actions and rewards are derived using the dynamics and prediction functions, which both rely on the use of a Monte Carlo tree search (MCTS) algorithm.

A MCTS allows a guided search of possible future actions. In brief, at a certain stage of the environment (such as a position in a game), subsequent possible actions and the resulting rewards are evaluated. Using these potential actions and rewards, a new hypothetical internal state is created and evaluated. Potential future states of *this* states are then considered and evaluated. This process occurs iteratively, forming expansive nodes off of the actual initial state, to allow for deeper search to evaluate more distant outcomes of potential actions. After expansion, the computed rewards are back-propagated up the tree to the current state and the mean value of an expansion is computed. Only then does the agent choose the optimal move or action and progress to the next state.

Unlike AlphaGo, in which the rules of the game are known, MuZero learns the mechanics of the game and environment, and can relatively reliably only expand nodes that will likely result in rewarding actions. This expansion is first done using the dynamics function, progressive states are mapped, in a reoccurring process, based on different possible action choices. At each hypothetical state, the function computes a reward and a potential new internal hidden state (the future environment). Both the reward and the new state are computed based on the previous hidden state (Schrittwieser et al., 2020).

Following the creation of each new state in muZero, a prediction function computes potential actions (a policy) and rewards (value) (Schrittwieser et al., 2020). The policy represents how the agent might act in this hidden state, while the value is an estimate of future rewards based on the current state. The value function is updated at each iteration of the dynamics function, as discussed.

This iterative process, of predicting and evaluating future actions in an environment has multiple corollaries in hippocampal processing: predictive place cell coding (the “successor representation”), theta sequences, and replay (Figure 1).

### Place cells and the successor representation

In an animal model, it is possible that place cell coding plays a predictive role in cognition: in the “successor representation” (SR) view of place cells, place cells encode features of the environment in relation to their predictive relationship with other features, including the potential presence of reward or potential future trajectories (Stachenfeld et al., 2017; Gershman, 2018). The SR view of place and grid cells explain the aforementioned bias toward significant features in coding. This view would also explain why cells in the HPC and EC maintain stable environmental representations in environments that vary in small ways that do not change the predictive value of contextual clues. In noisy or partially observable environments, models of SR place cells function better than pure cognitive map place cell models. Additionally, in the SR model, state relationships can be learned latently, while reward values are learned locally, allowing for fast updating of changes in reward structure in different contexts (Stachenfeld et al., 2017; Gershman, 2018; Vértes and Sahani, 2019).

### Theta sequences

The HPC also has an additional “lookahead” mechanism to evaluate future trajectory choices after an animal has experienced an environment and formulated a “map” with place cells. This hippocampal phenomenon, termed theta sequences, is a time-compressed and sequential firing of HPC place cells as if the animal were traversing a trajectory (Foster and Wilson, 2007). Because theta sequences can represent future possible trajectories, they are believed to play a role in planning (Wikenheiser and Redish, 2015a,b). Consistent with this idea, theta sequences incorporate information about future goal locations, and can use phase coupling of these rhythms to coordinate processing with other brain areas, much like the prediction function in muZero (Wikenheiser and Redish, 2015b; Penagos et al., 2017) (Figure 1).

In addition, the eHPC work in coordination with other structures to link present and future states with potential future actions. For instance, state prediction in HPC (*via* theta sequences) can be coupled through theta coordination coherence to other areas of the brain that process action and reward, including executive systems such as the prefrontal cortex (policy function) and dopaminergic areas like the VTA (reward function). Disruption of this coherence may, therefore, interrupt functions that require accurate predictions and may thus impact learning (Sigurdsson et al., 2010; Lesting et al., 2013; Igarashi et al., 2014).

### Replay

In muZero, sampling of future states occurs without the performance of an action and is most analogous to hippocampal offline processing that occurs during non-REM sleep and quiet wake. During these periods, cells in the hippocampus engage in replay: highly compressed (100s of ms) place cell sequences that form a trajectory (Wilson and McNaughton, 1994; Diba and Buzsaki, 2007). It is believed that hippocampal replay plays a fundamental role in memory consolidation and may also be important for planning (Gupta et al., 2010; O’Neill et al., 2010; Pfeiffer and Foster, 2013; Singer et al., 2013).

In one theory of hippocampal function, hippocampal replay also plays an important role in generalization by aiding in the offline construction of compressed environmental representations (Vértes and Sahani, 2019). During replay, events can be selectively sampled from visited event space, with priority given to representations that are reward predictive (Pfeiffer and Foster, 2013; Olafsdottir et al., 2015; Michon et al., 2019). Reward-predictive representations can then be compressed into a lower dimensional space that can be reused to plan future novel trajectories (Penagos et al., 2017). Replay may then use saved states to construct novel trajectories without the need for additional sampling or experience. Consistent with this idea, HPC replay selectively reinforces rewarded and significant locations, and it is possible that the shifting of place fields toward rewarded locations (a modification of representation) is dependent on replay. Additionally, replay can be comprised of novel sequences that have not been experienced, suggesting an environmental map can be generalized beyond prior experience (Gupta et al., 2010; Olafsdottir et al., 2015).

## Questions for context dependent learning

The means by which the HPC is involved in the differential encoding of separate spatial environments is well studied. This process, known as “remapping,” occurs when place cells change spatial representations in new environments (Colgin et al., 2008). In addition to the remapping that occurs when moving between starkly different environments, when exposed to continuous environmental change, place cells will gradually morph to remap to the new environment (Leutgeb et al., 2005). Although modeling experiments have shown that no threshold for remapping exists (Sanders et al., 2020), it is still unknown, experimentally if there is a threshold level of difference required to induce remapping between environments.

Importantly, learning also does not appear to be fully transferred between different environments where remapping is presumed to occur. For example, learning in both classical and operant conditioning tasks is nearly always context dependent, although the conditioned or operant response can be “re”-learned more quickly in the second environment (Urcelay and Miller, 2014). However, environmental changes may not affect the performance of navigational tasks to the same extent (Jeffery et al., 2003). Although cells in the HPC can encode conditioned and operant stimuli (as well as spatial location) (McEchron and Disterhoft, 1999; Wirtshafter and Wilson, 2019), it is not yet known whether any representation of the learned stimuli exists between remapped environments. Interestingly, however, animals with hippocampal lesions will transfer associative conditioning tasks across different environments (Penick and Solomon, 1991). It is similarly unknown whether this “failure” of context-dependent learning is due to an inappropriate preservation of stimuli encoding, or a remapping failure in which the representation of the first environmental interferes with the representation of the second.

Although muZero doesn’t explicitly perform context discrimination, using the model it has built of the environment, muZero must infer, probabilistically, whether enough contextual elements have changed to require a new map and, potentially, new learning. Studying how probabilistic inference gives rise to context independent learning in muZero may provide insight into human and animal learning and generalization. Establishing what rules muZero uses to determine when a new map is needed and when learning is context dependent may shed light on the mechanisms behind contextual learning in animals.

## Conceptual equivalence can result in predictions about hippocampal function

How might the advances made by muZero direct brain research? The evaluation of how transitions in cell representations occur between similar and distinct contexts is an under-researched topic in hippocampal systems neuroscience, and can be informed by advances in artificial intelligence agents such as muZero.

As previously explained, we believe AI can shed important light on the processes involved in contextual generalization. In muZero, there is a representational bias toward significant cues, and then this biased representation is compressed into a lower dimensionality. Representational bias allows for dimensionality reduction by beginning the selection of the most significant contextual elements (Lehnert et al., 2020). This reduction enables generalization by allowing learning inferences between environments with similarly compressed maps; i.e., in muZero, carry over of biased compressed representations allows for generalization (Lehnert et al., 2020).

In muZero, compression, which occurs during the dynamics function, plays an essential role in the generalization of learning. As previously explained, compression in the hippocampus may occur during hippocampal replay during sleep (Figure 1) (Penagos et al., 2017), and, like in muZero, this compression may be an important component of learning generalization (Vértes and Sahani, 2019). We might expect, therefore, for disruptions in sleep replay to cause deficits in task generalization (Hoel, 2021), and for increased replay (such as sleep sessions between tasks) to enhance learning generalization (Djonlagic et al., 2009). If replay plays an important role in generalization, we also might expect replay sequences and structure to be similar between two environments with the same task rules. Specifically, replay may be structured around task organization and reward-contingent actions, resulting in a clustering of replay around shared action states between two environments.

The compression of salient cues informs the creation of new hidden states, which may explain mechanisms of remapping between environments (Sanders et al., 2020). muZero’s dynamics function does more than calculate a new hidden state based solely on known context; it also makes inferences based on policy (action) and value (reward) mapping. In other words, a state representation not only reflects the environment, but also the actions that lead to environmental consequences. If the hippocampus follows a similar mechanism, it is likely that “knowing” when to remap requires more than a simple evaluation of environmental cues (there may be similar contexts that require different actions) but also necessitates identifying differences in potential policies and rewards, especially during periods of uncertainty (such as when experiencing novelty) (Penagos et al., 2017; Sanders et al., 2020; Whittington et al., 2020). Because a portion of this evaluation of state similarity and difference happens offline, sleep replay may be an essential mechanism for remapping. In this context, remapping may result in the generating or updating of hidden states when there is uncertainty due to change in inputs.

The speed and type (partial or complete) of remapping that occurs may therefore depend on how past experience is evaluated offline during replay (Plitt and Giocomo, 2021). We might also, therefore, expect disrupting replay to change the speed at which remapping occurs, or whether it occurs at all. Failures in remapping would likely also impact the ability to distinguish different environments during learning, thereby causing inability to learn skills in a new environment, conflation of skills learned in different environments, and/or perseverance of skills in the incorrect environment.

The majority of current hippocampal research focuses on representational correspondence between neural activity and context, such as how the brain represents the spatial correlates of an environment through neuronal firing patterns. Absent from much of this research is a larger focus on questions of algorithmic correspondence between neuron activity and context, such as determining the required difference between two environments to induce remapping. We believe advances in AI agents will assist in shifting research toward the investigation of algorithmic correspondence, *via* providing frameworks and predictions (such the necessity for replay in contextual generalization), by which to investigate the expected link between state changes and neuronal firing. Agents such as muZero can aid in illuminating these prospective questions, as well as shedding light on potential expected answers.

## Data availability statement

The original contributions presented in this study are included in the article/supplementary material, further inquiries can be directed to the corresponding author.

## Author contributions

HW and MW: study conception and design and manuscript editing. HW: draft manuscript preparation. Both authors reviewed the results and approved the final version of the manuscript.
